# Machine learning for the prediction of amyloid PET positivity using plasma biomarkers, cognition, APOE genotype, and structural imaging

**DOI:** 10.3389/fnins.2026.1905974

**Published:** 2026-08-17

**Authors:** Thony Yan Liang, Xueting Cui, Micheal Adeyosoye, Luana Okino Sawada, Mercedes Cabrerizo, Rosie E. Curiel Cid, Armando Barreto, Naphtali Rishe, David A. Loewenstein, Ranjan Duara, Malek Adjouadi

**Affiliations:** 1 Department of Electrical and Computer Engineering, Center for Advanced Technology and Education, Florida International University, Miami, FL, United States; 2 Knight Foundation School of Computing and Info Sciences, Florida International University, Miami, FL, United States; 3 1Florida Alzheimer’s Disease Research Center, Gainesville, FL, United States; 4 Department of Psychiatry, Behavioral Sciences and Neurology, Miller School of Medicine, University of Miami, Miami, FL, United States; 5 Center for Cognitive Neuroscience and Aging, Miami, FL, United States; 6 Mount Sinai Medical Center, Wien Center for Alzheimer’s Disease and Memory Disorders, Miami Beach, FL, United States

**Keywords:** ADNI, Alzheimer’s disease, amyloid PET, explainable artificial intelligence, machine learning, MRI, plasma biomarkers, p-tau217

## Abstract

**Purpose:**

Machine learning to enable precise, non-invasive detection of cerebral amyloid-beta (Aβ) pathology by integrating cognitive assessments, plasma biomarkers, and structural neuroimaging.

**Methods:**

We developed an explainable multimodal machine-learning framework to predict amyloid PET visual read status using plasma biomarkers, cognitive assessments, APOE genotype, demographic variables, and structural MRI measurements. The development cohort consisted of 170 participants from the 1Florida Alzheimer’s Disease Research Center (ADRC) and 129 participants from the ADNI4 cohort. Machine-learning pipelines were evaluated using combinations of five classifiers and multiple feature-selection approaches with Bayesian hyperparameter optimization and stratified five-fold cross-validation. Model interpretability was assessed using SHapley Additive exPlanations (SHAP).

**Results:**

Ensemble methods consistently outperformed linear and distance-based classifiers. The optimal pipeline, XGBoost with mutual-information feature selection, achieved a mean AUC of 0.891 ± 0.048 and a recall of 0.84. SHAP analyses identified the plasma p-tau217/Aβ42 ratio as the most influential predictor, followed by plasma p-tau217, p-tau181, MMSE, and APOE ε4 status. Structural MRI variables provided complementary predictive information. ADNI4 evaluation yielded a mean AUC of 0.545, while training on ADRC and testing on ADNI4 achieved an AUC of 0.636 and an overall accuracy of 63%. Importantly, plasma p-tau217/Aβ42 and p-tau217 remained the dominant predictors across cohorts.

**Conclusion:**

Multimodal machine learning can predict amyloid PET status while providing biologically interpretable explanations. Plasma tau and amyloid-related biomarkers carried the strongest predictive signal, while cognition, APOE genotype, and MRI refined classification decisions. External validation highlighted the challenges of cohort heterogeneity and domain shift but demonstrated preservation of biologically meaningful biomarker relationships.

## Introduction

Amyloid positron emission tomography (PET) imaging remains a clinically accepted approach for identifying cerebral amyloid deposition and guiding diagnostic evaluation (Johnson et al., 2013). However, PET imaging is expensive, resource intensive, and not universally accessible. There is also a challenge in finding concordance between amyloid visual read and quantitative amyloid load (Asken et al., 2024; Guevara et al., 2026). These limitations have motivated growing interest in blood-based biomarkers and computational approaches to predict amyloid pathology using less invasive, more scalable methods.

Recent advances in plasma biomarker development have substantially improved the ability to detect Alzheimer’s disease-related pathology (Jack et al., 2018, 2024). Among blood-based biomarkers, phosphorylated tau, particularly p-tau217, is showing a strong association with amyloid burden and disease progression, along with excellent concordance with both amyloid PET imaging and cerebrospinal fluid biomarkers (Ashton et al., 2024; Janelidze et al., 2021; Milà-Alomà et al., 2022; Palmqvist et al., 2020). Plasma amyloid ratios such as Aβ42/Aβ40 add further diagnostic utility for identifying amyloid positivity, while p-tau181, GFAP, and NfL contribute complementary information on amyloid burden, astrocytic activation, and neurodegeneration (Karikari et al., 2020; Teunissen et al., 2022).

This study intends to show that although these biomarkers individually show promise, combining them with cognitive assessments, genetic risk factors, and structural neuroimaging may further improve the predictive performance for Aβ status determination in concordance with the expert visual read. Although the present study used visual read classifications, future studies may evaluate relationships between predicted probabilities and Centiloid (CL) measures (Klunk et al., 2015).

Machine-learning methods are well suited to integrating heterogeneous clinical, imaging, and biomarker data while capturing nonlinear relationships that conventional statistical approaches may miss and have shown increasing utility in neurodegenerative disease research (Jo et al., 2019; Vieira et al., 2017). Prior studies have demonstrated that ensemble and tree-based learning methods can effectively model interactions among biomarkers, cognition, and imaging variables for Alzheimer’s disease classification and progression prediction. However, many existing studies rely on limited modalities, small feature sets, or single-model approaches, reducing interpretability and generalizability. In addition, understanding which features consistently drive prediction remains critical for translating machine-learning systems into clinically meaningful decision-support tools.

Explainable artificial intelligence methods, such as SHapley Additive exPlanations (SHAP), provide a mechanism for interpreting complex machine-learning models by quantifying the contribution of each feature to model predictions (Lundberg and Lee, 2017; Lundberg et al., 2020). These approaches allow both global and subject-level interpretation, helping identify the biological and clinical variables most strongly associated with amyloid positivity while improving transparency of predictive systems.

The present study developed and evaluated a multimodal machine-learning framework for predicting amyloid-PET status using clinical, demographic, cognitive, plasma biomarker, and MRI-derived structural features. Multiple classifiers and feature-selection strategies were systematically compared within a rigorously cross-validated framework designed to minimize information leakage. In addition to evaluating predictive performance, this study aimed to identify the most influential predictors of amyloid positivity using SHAP-based explainability analyses. We hypothesized that tree-based machine-learning models integrating plasma biomarkers with MRI and cognitive measures would demonstrate strong predictive performance and that plasma tau- and amyloid-related biomarkers would emerge as dominant contributors to amyloid-PET classification.

## Materials and methods

The objective of this study was to develop and evaluate machine-learning models to predict amyloid-PET visual read outcomes, i.e., amyloid-positive or amyloid-negative. Features were drawn from four domains: Clinical, demographic, cognitive, and plasma biomarkers, as well as MRI volume and cortical thickness variables. In addition to prediction performance, the study aimed to identify the most informative features for characterizing amyloid status, with the broader goal of isolating the variables most relevant to amyloid detection. Amyloid PET visual read was used as the reference standard because it represents the clinically accepted method for determining amyloid positivity and was consistently available for all participants, whereas quantitative Centiloid measurements were not uniformly available across the complete study cohort.

### Data processing

To ensure that all modeling pipelines were evaluated on the same analysis sample, we restricted the dataset to participant visits with complete outcome and feature information. Characteristics of the participant’s dataset are seen in Table 1. This step helped maintain consistency across all models and prevented differences in sample size from influencing performance comparisons. For visits with PET data but missing MRI volumes, MRI values were recovered from the same participant’s nearest MRI visit only when the PET and MRI scans were within 12 months of each other; otherwise, the values remained missing and were later removed by the data-completeness filter. This approach allowed us to preserve as many visits as possible while avoiding MRI data that were too far removed in time from the PET scan to reflect the same underlying disease state.

**TABLE 1:** Demographic, cognitive, and biomarker characteristics of the study sample (*n* = 170).

Feature	Category	Value
Age [years, mean (SD)]		70.69 (8.03)
Sex [n, %]	Male	73 (42.9%)
	Female	97 (57.1%)
Race/ethnicity [n, %]	White, non-Hispanic	81 (47.6%)
	White, Hispanic	82 (48.2%)
	Black African American, non-Hispanic	5 (2.9%)
	Asian/Pacific Islander	1 (0.6%)
	Multiracial	1 (0.6%)
Education [years, mean (SD)]		15.59 (3.35)
Cognitive status [n, %]	NC	18 (10.6%)
	Impaired not SCD/MCI	8 (4.7%)
	SCD	29 (17.1%)
	EMCI	57 (33.5%)
	LMCI	29 (17.1%)
	Dementia	29 (17.1%)
MMSE [mean (SD)]		26.27 (4.86)
CDRSUM [mean (SD)]		2.19 (2.71)
APOE ε4 Alleles [n, %]	0	101 (59.4%)
	1	57 (33.5%)
	2	12 (7.1%)
Amyloid Aβ PET, status [n, %]	Negative	102 (60%)
	Positive	68 (40%)
Plasma Aβ40 pg/mL [mean (SD)]		206.55 (56.35)
Plasma Aβ42 pg/mL [mean (SD)]		8.56 (2.51)
Plasma p-tau217 pg/mL [mean (SD)]		0.69 (0.52)
Plasma p-tau181 pg/mL [mean (SD)]		28.34 (14.10)
Plasma GFAP pg/mL [mean (SD)]		189.31 (107.16)
Plasma NFL pg/mL [mean (SD)]		15.91 (12.87)

The cohort spanned a broad range of cognitive states that included cognitively normal (CN), impaired not subjective cognitive decline or mild cognitive impairment (Impaired Not SCD/MCI), subjective cognitive decline (SCD), early mild cognitive impairment (EMCI), late mild cognitive impairment (LMCI), and dementia. Impaired Not SCD/MCI refers to participants judged by clinical consensus to have subtle cognitive deficits, but whose performance did not meet formal criteria for subjective cognitive decline or mild cognitive impairment. This category is consistent with the Uniform Data Set version 3 (UDS3) (Besser et al., 2018) designation and aligns with the concept of pre-mild cognitive impairment (PreMCI) (Loewenstein et al., 2012). These categories reflect progressively later clinical stages along the Alzheimer’s disease continuum and provide important context for interpreting plasma biomarker variation. In general, individuals in later symptomatic stages, particularly those with Alzheimer’s disease pathology, tend to exhibit higher or more abnormal blood biomarker levels, especially tau-related measures such as plasma phosphorylated tau 181 and 217. MRI volumes were normalized by estimated total intracranial volume (eTIV) to account for inter-participant differences in head size. After normalization, eTIV was removed from the model. Blood biomarkers with coefficients of variation (CV) > 0.2 were excluded as part of quality control to reduce the influence of unreliable assays and improve the robustness of the downstream models. After this filtering step, two plasma biomarker ratios were derived: Aβ42/Aβ40 and p-tau217/Aβ42. These ratios were selected because previous studies have demonstrated that combining tau and amyloid biomarkers improves discrimination of amyloid burden relative to individual markers alone (Cullen et al., 2021; Palmqvist et al., 2020).

### Model pipeline design

**FIGURE 1:**
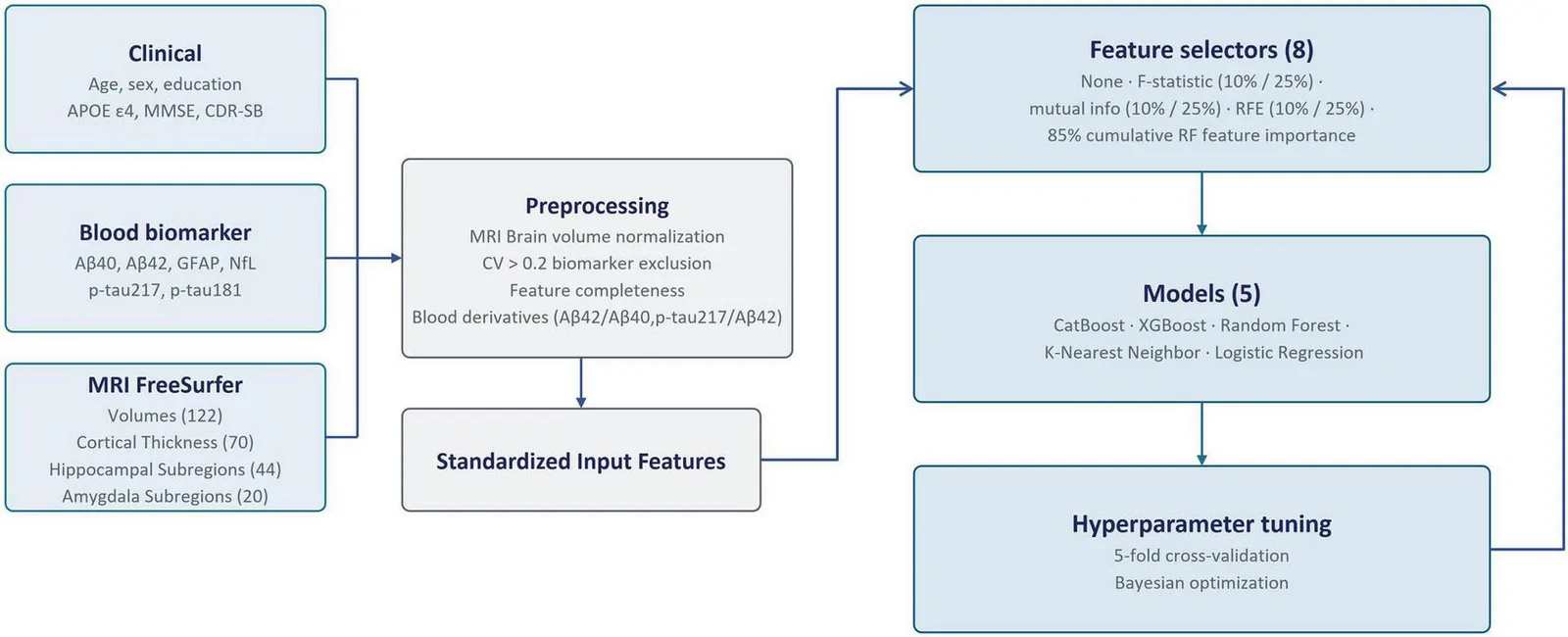
Overview of the machine learning pipeline. Input features were drawn from three domains: Clinical variables, plasma biomarkers, and MRI FreeSurfer measures.

To avoid relying on a single machine-learning model, we evaluated a range of classification methods alongside multiple feature-selection strategies, as seen in Figure 1, consistent with the pipeline structure described by Karlsson et al. (2025) and Mattsson-Carlgren et al. (2026). Each model was implemented as a scikit-learn pipeline consisting of standardization, an optional feature-selection step, and a classifier. Standardization was performed within the cross-validation loop rather than on the full dataset, which helped reduce the risk of information leakage and preserved the integrity of model evaluation.

### Hyperparameter tuning

For each configuration, classifier hyperparameters were optimized via Bayesian search with stratified 5-fold cross-validation and recall as the objective. The search spaces were tailored for each classifier to include the parameters most relevant to its performance, as seen in Table 2. Logistic regression was tuned over regularization strength and solver choice. Random forest over tree depth and number of estimators. XGBoost over tree depth, learning rate, regularization, and number of estimators. K-nearest neighbors over the number of neighbors. A fixed iteration budget was assigned to each classifier, with more complex models allowed additional search iterations, to balance thorough tuning with computational efficiency.

**TABLE 2:** Bayesian hyperparameter search configuration for each classifier.

Classifier	Hyperparameters tuned	Search space	Iterations
Logistic regression (LR)	*C*, solver	*C* ∈ [10^−4^,10^3^] log-uniform; solver ∈ {lbfgs, saga}	20
Random forest (RF)	Max depth, n estimators	max_depth ∈ [2,12]; n_estimators ∈ [5,500]	50
XGBoost (XGB)	max depth, learning rate, reg_lambda, reg_alpha, n_estimators	max_depth ∈ [2,12]; eta ∈ [0.01,0.3] log-uniform; reg_lambda ∈ [10^−8^,1] log-uniform;reg_alpha ∈ [10^−8^,1] log-uniform; n_estimators ∈ [5,500]	100
K-nearest neighbors (KNN)	n_neighbors	n_neighbors ∈ [3,26]	20
CatBoost (CatB)	depth, n_estimators, learning_rate	depth ∈ [2,12]; n_estimators ∈ [5,500]; learning_rate ∈ [0.01,0.3] log-uniform	40

The table lists the hyperparameters included in the search, their corresponding search spaces, and the number of optimization iterations used during Bayesian optimization.

### Cross-validation and feature selection

Model performance was estimated using a separate stratified five-fold cross-validation procedure with the tuned hyperparameters. For each fold, four partitions were used for training and one for testing, and all preprocessing steps were fit only on the training partition. Feature selection was performed independently within each fold using only that fold’s training data, and the resulting selected feature subset was then applied to the held-out test fold. In other words, the test fold did not influence which features were selected for that round of training. For recursive feature elimination (RFE), the recursive elimination process repeatedly removes the least useful features from the training data until the target number of features is reached. For RF-based selection, the random forest was fit on the training partition, feature importance scores were computed from that training data only, and the selected subset was passed to the downstream classifier. This fold-wise procedure was repeated across all five splits, which allowed you to record how often each feature was selected and identify features that appeared consistently across folds. That frequency-based summary provides a measure of stability rather than a single global ranking.

### Computational platform and processing time

The pipeline was run on an NVIDIA DGX H100 server that is equipped with dual Intel Xeon Platinum 8,480 C processors and 2TB of system RAM. All computations were executed inside a Docker container on the system. Table 3 shows the processing time for each pipeline.

**TABLE 3:** Processing time (in seconds) for each combination of feature selection method and classification model.

Model Feature selector	LR	KNN	RF	XGBoost	CatBoost
None	33.7	35.0	208.6	844.8	5,105.6
RFE10	124.5	131.3	504.2	995.2	1,751.2
RFE25	100.6	116.7	495.8	990.1	1,284.2
RF Importance	46.3	54.0	275.7	862.0	1,910.6
f_perc10	21.9	38.8	191.8	635.7	1,025.2
f_perc25	22.9	37.1	136.5	694.7	1,240.3
mir_perc10	61.1	75.4	365.3	880.6	849.1
mir_perc25	64.2	75.8	333.3	883.9	1,531.0

### Performance evaluation

After fitting each fold-specific pipeline, predictions were generated on the held-out test fold, and performance was summarized using F1, accuracy, precision, and recall. These metrics were then averaged across five folds and reported as mean and standard deviation for each configuration. Configurations were ranked by mean recall to identify the most effective pipeline for the prediction task, as recall directly reflects sensitivity to the positive class and therefore prioritizes minimizing false negatives. This framework allowed direct comparison across models while keeping training, feature selection, and evaluation properly separated.

### Model interpretation with SHAP

To interpret the selected model beyond aggregate performance metrics, SHAP values were computed only on the test dataset for each fold. This was done to avoid leakage between the data used for model training and the data used to explain predictions. For each fold, the fitted preprocessing pipeline was applied to the test data, and SHAP values were computed in the same transformed feature space that the estimator received. This is important because the explainer must operate on the exact representation used by the model.

Because the best-performing classifier was XGBoost, tree-based SHAP was used to compute exact Shapley values efficiently from the tree structure. SHAP values were obtained on the model’s raw margin output, and predicted probabilities were derived by sigmoid transformation. For each participant, the recorded outputs included the fold identifier, participant identifier, true label, predicted label, predicted probability, per-feature SHAP values, expected value, and retained feature names. These outputs supported both global interpretations, through aggregation of absolute SHAP values across participants, and local interpretation, through inspection of individual predictions.

### Assay and PET imaging

For the 1Florida Alzheimer’s Disease Research Center (ADRC) cohort, plasma Aβ42, Aβ40, and p-tau181 concentrations were quantified using the Simoa Human Neurology 3-Plex A assay (Quanterix, Billerica, MA, United States). Plasma glial fibrillary acidic protein (GFAP) and neurofilament light chain (NfL) levels were measured using the Simoa Human Neurology 2-Plex B assay. Plasma p-tau217 concentrations were determined using the ALZpath p-tau217 assay, a highly sensitive immunoassay developed for the detection of Alzheimer’s disease–related tau pathology. Amyloid PET imaging was performed using florbetaben F-18 (Neuraceq^®^), an FDA-approved radiotracer that binds to fibrillar β-amyloid plaques and enables *in vivo* assessment of cerebral amyloid burden. Trained readers interpreted PET scans according to established clinical guidelines, and amyloid status was classified as positive or negative based on expert visual assessment.

For the ADNI4 cohort, plasma Aβ42, Aβ40, and p-tau217 were measured using fully automated Fujirebio Lumipulse immunoassays, whereas GFAP and NfL concentrations were quantified using Quanterix Simoa assays. These biomarker measurements were generated through the ADNI Biomarker Core to support standardized blood-based assessment of Alzheimer’s disease pathology across participating sites.

## Results

**FIGURE 2:**
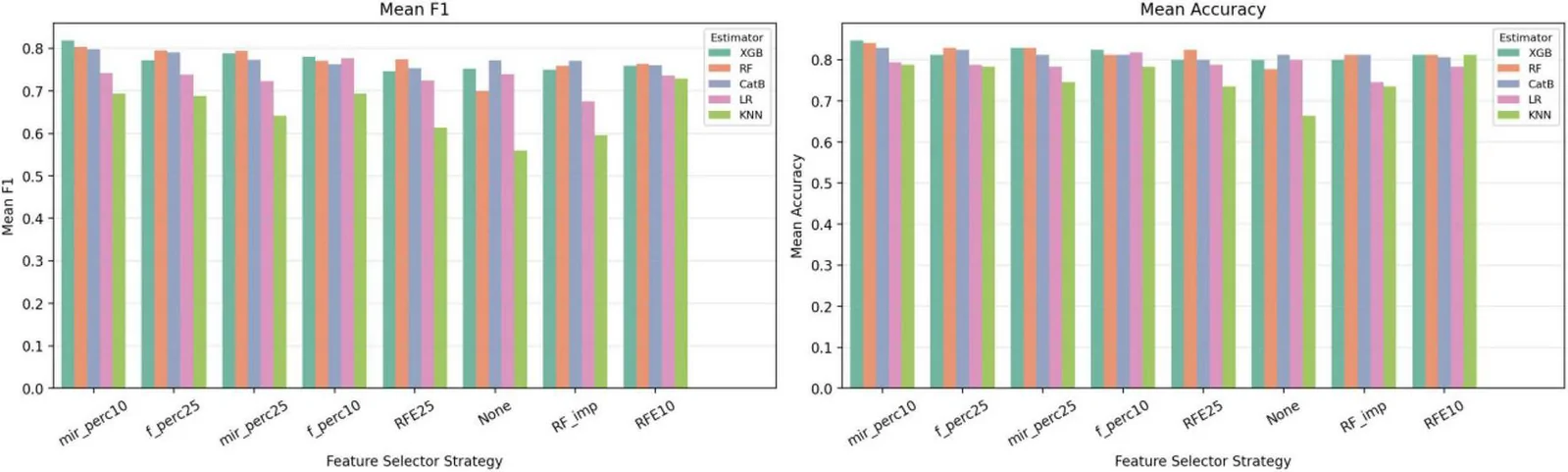
Mean F1 (left) and mean accuracy (right) for all classifier and feature-selector combinations in the 1FL ADRC cohort.

**FIGURE 3:**
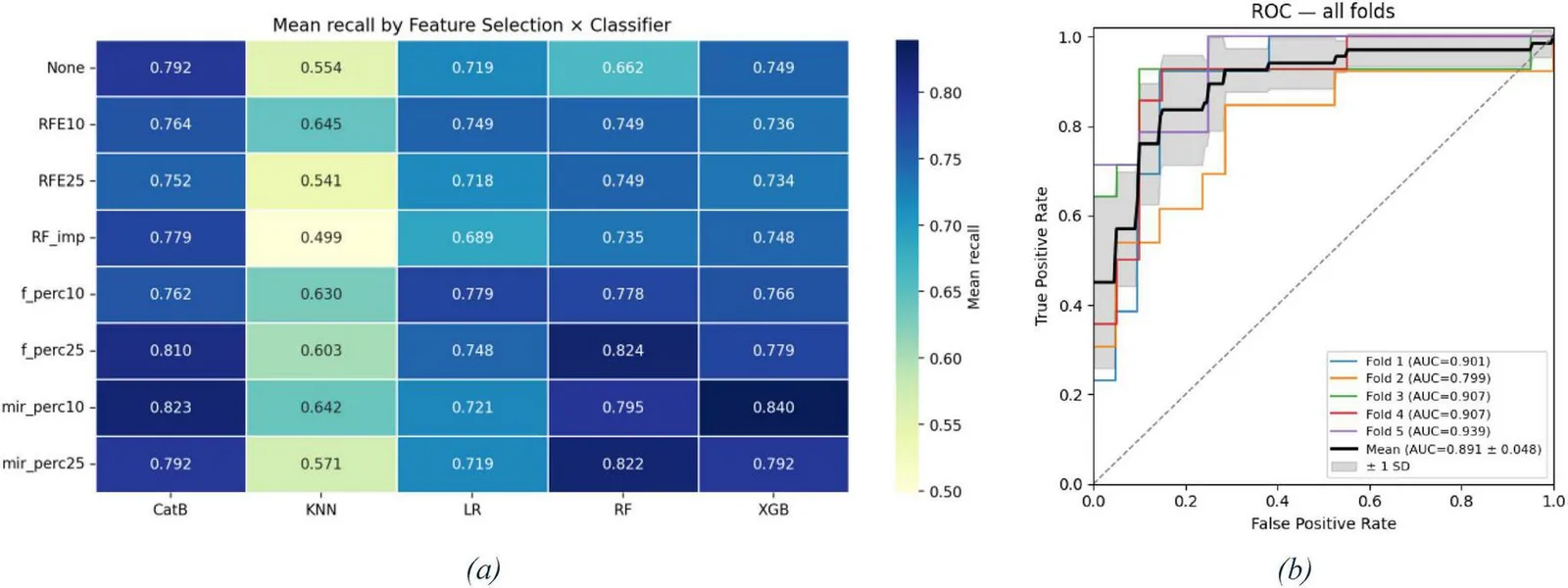
1FL ADRC ML pipeline results. **(a)** Heatmap of mean recall for all model pipelines. **(b)** ROC curves per fold for the mutual-information selector at 10% retention with XGBoost.

Across the evaluated machine learning pipelines, tree-based methods outperformed linear and distance-based approaches Figure 2. The heatmap of mean recall across all pipelines showed that the best-performing pipeline was the XGBoost model with mutual information feature selection at 10% feature retention, achieving a mean recall of 0.84, as seen in Figure 3a, while strong competing configurations included random forest with F-test at 25% retention and CatBoost with mutual information feature selection at 10% feature retention. This pattern was largely consistent with the F1 and accuracy plots, in which XGBoost, CatBoost, and random forest showed superior, relatively stable performance across multiple feature-selection settings. In contrast, k-nearest neighbors showed weaker, more variable results. These findings suggest that the prediction task benefits more from nonlinear modeling and flexible interaction learning than from simpler decision boundaries.

**FIGURE 4:**
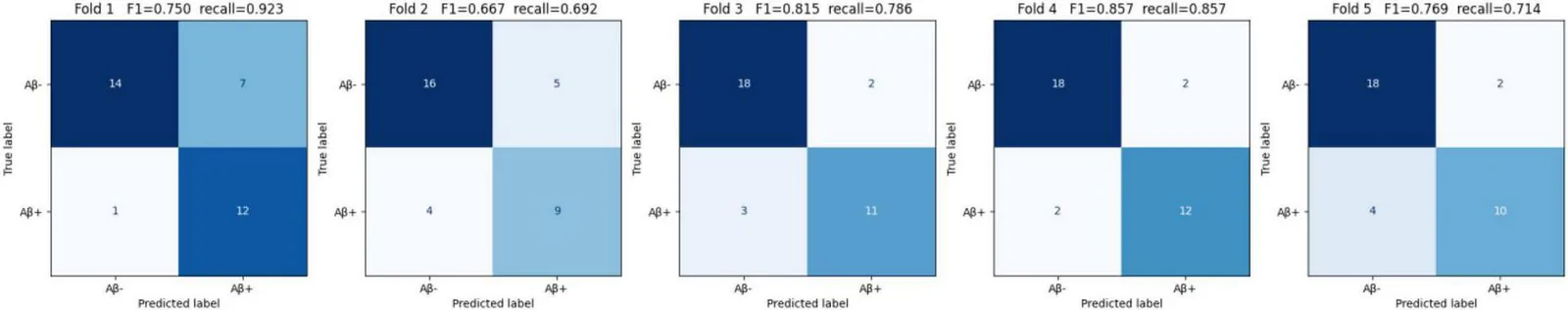
Confusion matrices across the five cross-validation folds, with fold-specific F1 and recall.

The receiver operating characteristic (ROC) curve analysis further supported the pipeline’s robustness. Across the five cross-validation folds, the area under the curve (AUC) had a mean of 0.891 and a standard deviation of 0.048 in seen Figure 3b. Although one-fold performed worse than the others, the remaining folds demonstrated consistently high predictive performance, indicating that the model generalized reasonably well across different train-test splits rather than relying on a single favorable split. The confusion matrices showed fold-specific F1 scores ranging from 0.667 to 0.857, as seen in Figure 4, again indicating good but imperfect classification performance, with a small number of both false-positive and false-negative cases persisting in each fold.

**FIGURE 5:**
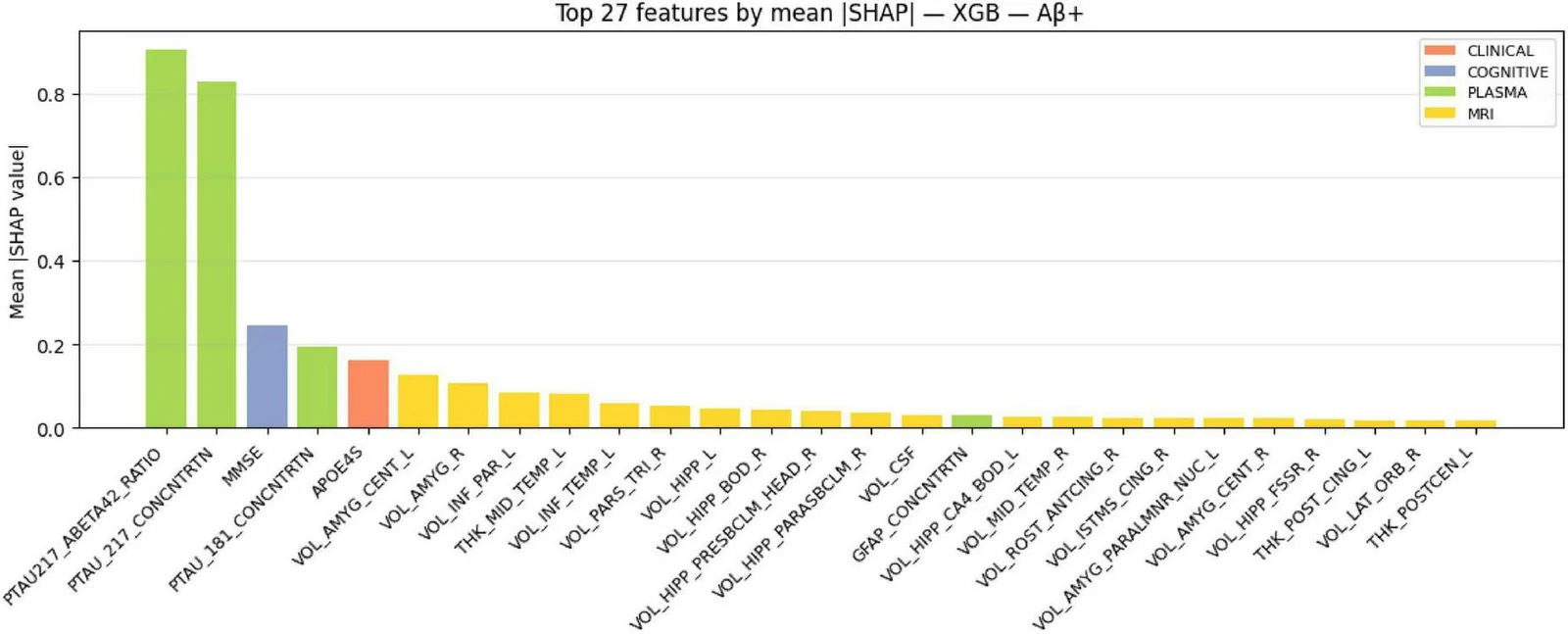
Top 27 features by mean absolute SHAP value for the mutual-information selector at 10% retention with XGBoost model predicting Aβ+, color-coded by domain: clinical, cognitive, plasma, and MRI.

SHAP model analyses indicated that plasma biomarkers were the dominant drivers of prediction. The prominence of p-tau217-based measures observed in the present study is highly consistent with previous multicenter investigations demonstrating superior diagnostic performance of plasma p-tau217 compared with other circulating biomarkers for identifying amyloid-positive individuals (Hansson et al., 2023; Palmqvist et al., 2020). In the mean absolute SHAP ranking, the p-tau217/Aβ42 ratio was the most influential feature, followed closely by plasma p-tau217 concentration, while MMSE and plasma p-tau181 provided additional but smaller contributions. APOE ε4 status also emerged as one of the leading predictors, and several MRI regional volume measures contributed modestly to the model, indicating that structural imaging added complementary information beyond blood biomarkers alone, as shown in Figure 5.

The results in Figure 5 demonstrate that plasma biomarkers dominated the predictive hierarchy, substantially exceeding the influence of imaging and demographic variables. The most influential feature was the plasma p-tau217/Aβ42 ratio, indicating that the combined relationship between phosphorylated tau pathology and amyloid burden carried the strongest discriminative signal for identifying Aβ positivity. Elevated p-tau217 reflects tau-related neurodegeneration associated with amyloid pathology, while reductions in plasma Aβ42 are associated with cerebral amyloid deposition. Similarly, plasma p-tau181 also ranked highly, although with a smaller contribution than p-tau217, consistent with prior reports suggesting that p-tau217 may outperform p-tau181 for early amyloid detection and disease staging. The box plots of our own 1Florida ADRC data support this assertion.

Among non-biomarker variables, MMSE and APOE ε4 status demonstrated important contributions to classification performance. MMSE likely reflects downstream cognitive manifestations of underlying pathology, whereas APOE ε4 status is an established genetic risk factor for amyloid accumulation.

The relatively lower contribution of MRI variables is biologically plausible because structural atrophy often reflects downstream neurodegenerative processes that occur after the emergence of amyloid pathology. Similar observations have been reported in previous multimodal biomarker studies, where molecular biomarkers outperform structural imaging for amyloid classification tasks (Frisoni et al., 2024; Ossenkoppele et al., 2021, 2025).

These results support that amyloid-PET status can be predicted with good accuracy using a multimodal machine-learning framework, but that the strongest signal is carried by plasma-based tau and amyloid measures. Cognitive performance, genetic risk, and structural MRI features appear to refine prediction rather than define it. This suggests that these modalities provide context in addition to the core blood biomarker signal. The strong performance of plasma features is encouraging, suggesting that blood-based biomarkers can capture much of the signal associated with amyloid pathology, with MRI and clinical variables.

**FIGURE 6:**
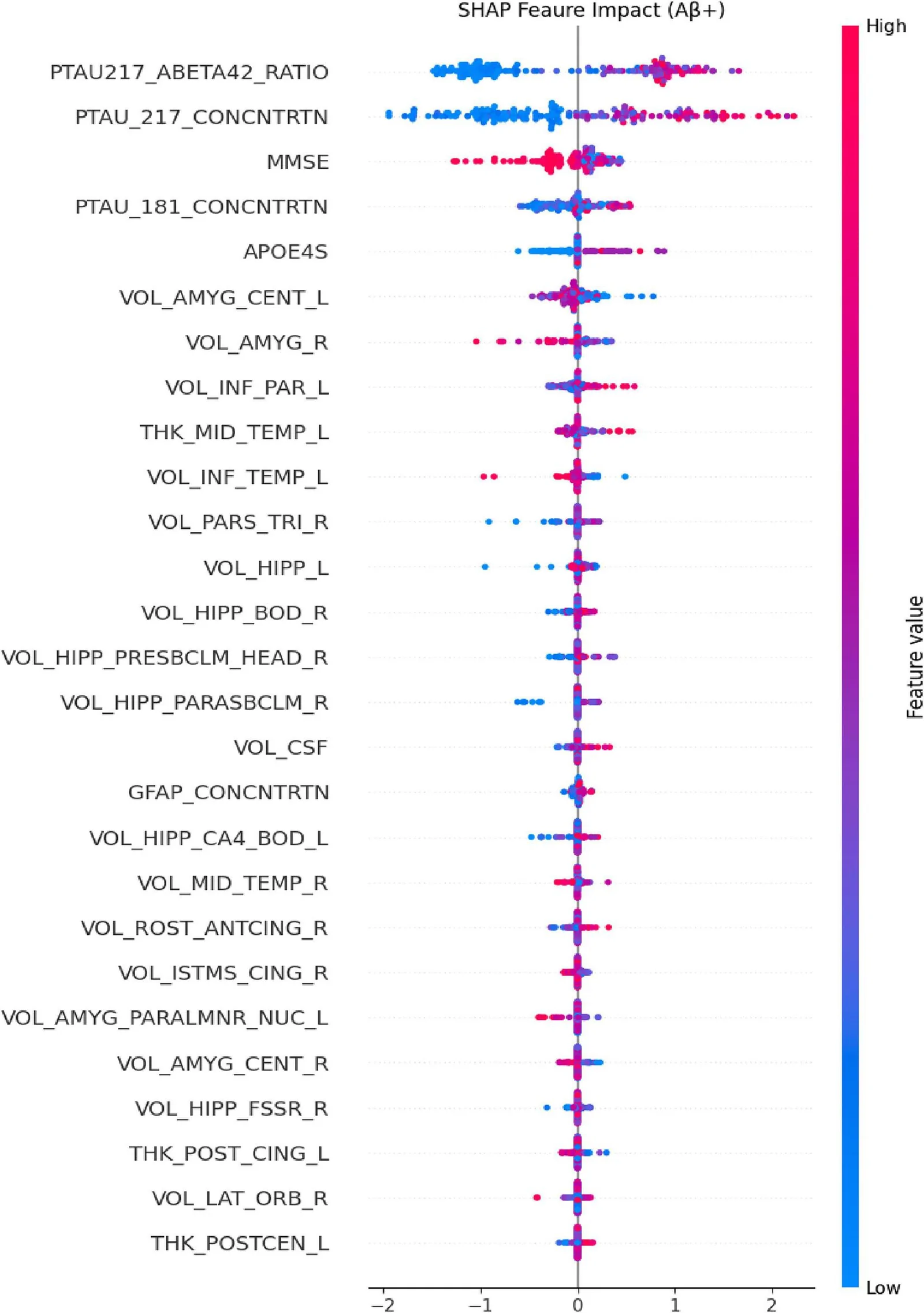
Top 27 Features for SHAP Feature impact toward Aβ+.

**FIGURE 7:**
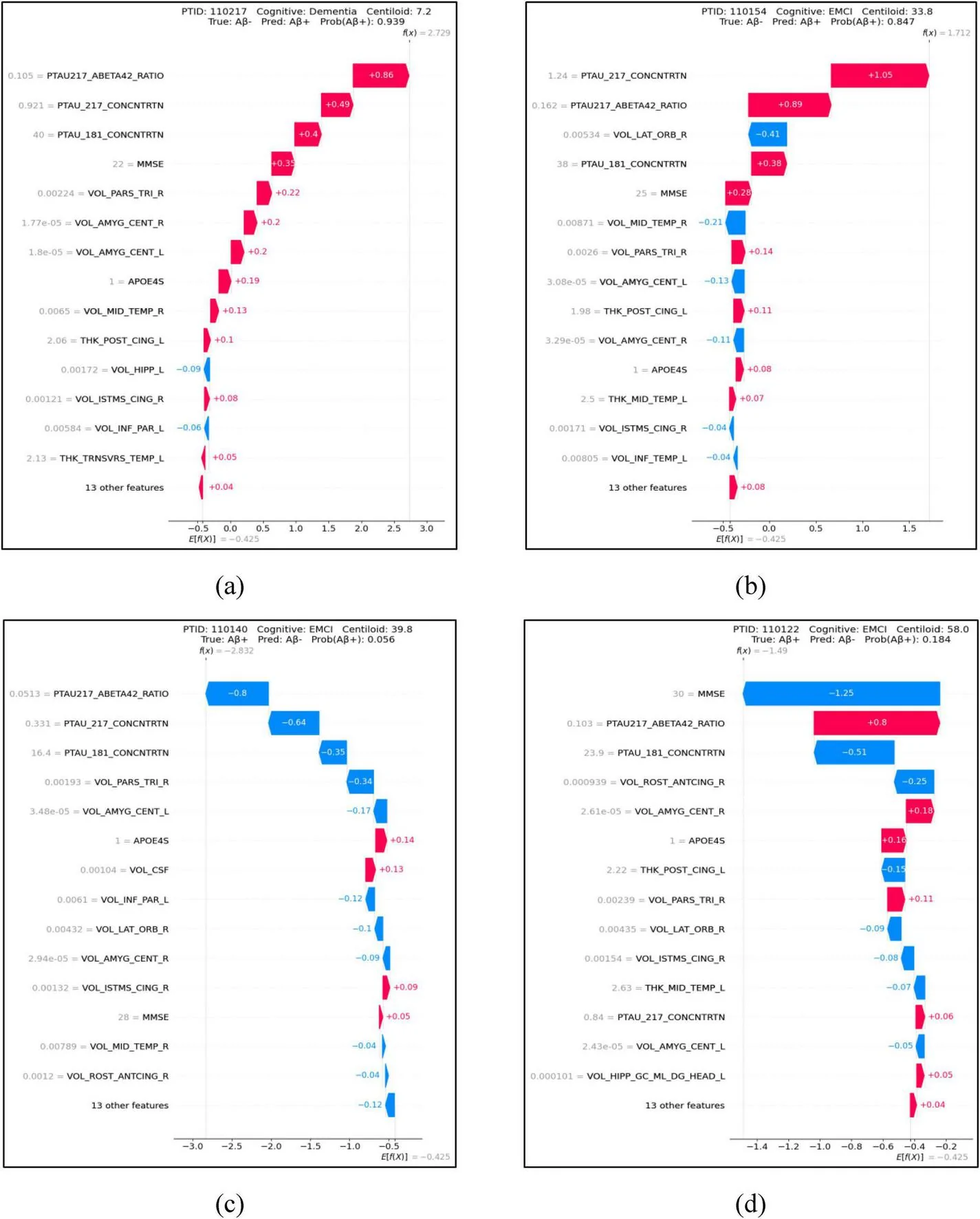
SHAP values for individual prediction. Positive SHAP values move the prediction toward amyloid positivity **(a,b)**, whereas negative SHAP values shift it toward amyloid negativity **(c,d)**. Ordered by how each feature influences the prediction.

The SHAP beeswarm plot in Figure 6 shows the top 27 features contributing to model predictions and illustrates both the magnitude and directionality of each feature’s impact on Aβ classification. This SHAP beeswarm plot reinforced this hierarchy and showed that higher values of the leading plasma markers generally shifted predictions toward the amyloid-positive class. In contrast, lower values shifted them toward the negative class. Unlike the global ranking shown in Figure 7, the beeswarm plot provides participant-level variability and demonstrates how high or low feature values influence predictions toward either Aβ-positive or Aβ-negative classifications.

The beeswarm plot thus reinforces the dominant importance of plasma biomarkers observed in the global SHAP ranking. Features such as the p-tau217/Aβ42 ratio, plasma p-tau217, and p-tau181 consistently displayed the largest SHAP magnitudes, indicating that these variables exerted the strongest influence on model predictions across participants. High values of these biomarkers generally shifted predictions toward the Aβ-positive class, whereas lower values shifted predictions toward the Aβ-negative class. This directional consistency strengthens the model’s biological plausibility because elevated phosphorylated tau concentrations are strongly associated with Aβ status.

### Error analysis

To better understand model behavior, we examined individual predictions using local SHAP explanations and the corresponding Centiloid values for the misclassified cases shown in Figure 7. This analysis was based on the best-performing cross-validation fold and therefore reflects the fold-level examples rather than average behavior across all folds. The selected cases were incorrect predictions, but they are still useful for understanding how the model integrates plasma biomarkers, cognition, APOE status, MRI-derived features, and quantitative amyloid burden when making borderline decisions.

The addition of Centiloid data helps contextualize these misclassifications, because Centiloid provides a standardized measure of amyloid burden and is commonly interpreted along a continuum rather than as a strict binary label. Values below 10 CL are generally considered negative, with values above 30 CL considered positive, and the interval in between is often treated as an intermediate or gray zone (Iaccarino et al., 2025). Since these thresholds are conventions and Centiloid carries measurement variability, values close to either boundary are best interpreted cautiously rather than definitely negative or positive by visual read.

In Figure 7a, the model predicted amyloid positivity with high probability despite the true label being amyloid-negative. The main drivers were strong positive contributions from the p-tau217-related features, including the p-tau217/Aβ42 ratio, p-tau217, and p-tau181, together with MMSE, which together outweighed the smaller opposing effects from MRI variables such as hippocampal and temporal measures. This case illustrates how a blood biomarker profile that strongly resembles an amyloid-positive pattern can dominate the prediction, even when the visual read is negative. Although 7.2 CL falls below the 10 CL negative threshold, it sits close to the lower edge of the intermediate range. Given that Centiloid measurement variability, this supports the possibility of a low-but-nonzero amyloid burden rather than a truly discordant biological profile.

Figure 7b showed a similar false-positive pattern, although with slightly lower confidence. In this case, the blood biomarkers again pushed the prediction toward amyloid positivity, while MRI-derived measures exerted counterbalancing effects toward amyloid negativity. At 33.8 CL, the value lies just above the 30 CL positivity threshold, near the upper edge of the intermediate range, suggesting the participant may have occupied a borderline region in which the prediction did not fully capture continuous amyloid burden. This pattern indicates that the model was integrating competing multimodal signals rather than relying on a single feature in isolation.

In Figures 7c,d, the model predicted amyloid negativity despite true amyloid-positive labels. In both cases, the decision was driven primarily by the same top predictors seen in the global SHAP ranking, especially p-tau217, the p-tau217/Aβ42 ratio, p-tau181, and MMSE, but with different directional effects across subjects. In one case, lower biomarker values and relatively preserved cognition favored amyloid negativity, whereas in the other, opposing APOE and MRI contributions were insufficient to overcome the negative biomarker profile. However, the Centiloid values were around 40 or higher, indicating amyloid burden and supporting the interpretation that these were not borderline cases but rather amyloid-positive individuals whose visual read was not fully captured by the local model output. This suggests that the apparent misclassification reflects model disagreement with the visual read rather than absence of meaningful amyloid pathology.

Overall, the error analysis suggests that misclassifications often arose from biologically meaningful borderline cases rather than random model failure. The addition of Centiloid values strengthens this interpretation by showing that some of the apparent errors may reflect the limitations of amyloid visual read in representing a continuous pathological spectrum. Thus, the model appears to capture meaningful amyloid-related biology, especially through plasma tau and amyloid markers. At the same time, Centiloid values help place the local explanations in a quantitative disease-burden context. These findings support the value of explainable artificial intelligence in identifying clinically informative transitional states and understanding how multimodal features jointly shape predictive decisions.

### Sex stratified performance

Sex-related biological variability is a potential source of error in biomarker-based prediction models, particularly because prior work suggests that both biological sex and sampling diversity can account for meaningful variance in neurodegeneration-related measures (Abu Raya et al., 2025; Rudroff, 2026). In a sex-stratified evaluation across the five cross-validation folds in the 1FL ADRC cohort, the mean male AUC was 0.962 ± 0.039. In contrast, the mean female AUC was 0.847 ± 0.110, indicating higher average performance in males but also greater stability across folds. The wider fold-to-fold variation in the female subgroup suggests that subgroup-specific uncertainty may contribute to overall model variability even when the same model is trained on the full mixed-sex cohort. At the same time, sex was available among the candidate clinical variables. However, it was not selected by the final feature-selection procedure in any fold, indicating that sex itself was not retained as a dominant direct predictor in the winning model.

Sex-stratified SHAP analysis nevertheless suggested broad agreement rather than divergence in the dominant attribution structure across sexes. The five highest-ranked features were the same in both groups, namely the plasma p-tau217/Aβ42 ratio, plasma p-tau217, MMSE, plasma p-tau181, and APOE ε4 status, and the directional effects of the leading plasma biomarkers were preserved, with higher p-tau217 and higher p-tau217/Aβ42 values shifting predictions toward amyloid positivity in both groups. Differences in mean absolute SHAP values were largely confined to lower-ranked features. They were small relative to the leading biomarkers, suggesting that the model’s core predictive logic remained stable across sex. Together, these findings suggest that sex-related variability in this cohort was more evident in predictive performance and stability than in the ranking or directional behavior of the dominant predictive features.

### ML pipeline on ADNI

To evaluate external generalizability, the optimized XGBoost framework developed using the 1Florida ADRC cohort was assessed on an independent ADNI4 cohort comprising 129 participants. The ADNI4 population differed substantially from the ADRC cohort, with a higher proportion of cognitively normal individuals (66.7% vs. 32.4%) and a larger fraction of amyloid-negative participants (64% vs. 60%), creating a substantial distributional shift between cohorts.

Based on ADNI4 cohort statistics, the distribution of cognitive diagnosis stages differed from that of the 1FL ADRC training cohort, as shown in Table 4. Even after harmonizing cognitive diagnosis categories from 1FL ADRC, the distribution remained distinct, as shown in Table 5. In particular, the relatively large proportion of cognitively normal participants in ADNI4 likely contributed to a different distribution of plasma biomarkers compared with that in 1FL ADRC. These differences suggest that the ADNI4 dataset was structurally dissimilar to the 1FL ADRC cohort, which may have limited the machine learning pipeline’s ability to generalize. In addition, missing values in ADNI4 (*n* = 62) were handled using a KNN imputer with *k*5, applied to a predictor set that included clinical variables such as age, sex, education, APOE4S, CDRSUM, and MMSE, as well as plasma biomarker variables and biomarker-derived ratios. The missing values were most commonly GFAP and NfL measurements, likely reflecting a change in assay platform.

**TABLE 4:** ADNI KNN imputed statistics, GFAP, and NFL blood biomarkers are the only imputed values, meaning all other feature statistics are correct (*n* = 129).

Feature	Category	Value
Age [years, mean (SD)]		71.86 (7.97)
Sex [n, %]	Male	56 (43.4%)
	Female	73 (56.6%)
Race/ethnicity [n, %]	White, non-Hispanic	71 (55.0%)
	White, Hispanic	17 (13.2%)
	Black African American, non-Hispanic	32 (24.8%)
	Asian/Pacific Islander	5 (3.2%)
	Multiracial	4 (0.6%)
Education [mean (SD)]		16.50 (2.53)
APOE ε4 alleles [n, %]	0	90 (69.8%)
	1	32 (24.8%)
	2	7 (5.4%)
MMSE [mean (SD)]		28.37 (1.96)
CDRSUM [mean (SD)]		0.51 (0.91)
Diagnosis [n, %]	CN	86 (66.7%)
	MCI	39 (30.2%)
	Dementia	4 (3.1%)
Amyloid Aβ PET status [n, %]	Negative	83 (64%)
	Positive	46 (36%)
Plasma Aβ 40 pg/mL [mean (SD)]		317.63 (62.56)
Plasma Aβ 42 pg/mL [mean (SD)]		27.32 (5.99)
Plasma p-tau217 pg/mL [mean (SD)]		0.20 (0.21)
Plasma GFAP pg/mL [mean (SD)]		162.99 (99.91)
Plasma NfL pg/mL [mean (SD)]		16.91 (14.47)

**TABLE 5:** ADNI and 1FL ADRC Diagnosis matching.

Harmonized cognitive status	ADNI categories	ADNI (n)	1FL ADRC categories	1FL ADRC (n)
Cognitively normal (CN)	CN	86	NC, impaired not SCD/MCI, SCD	55
Mild cognitive impairment (MCI)	MCI	39	EMCI, LMCI	86
Dementia	Dementia	4	Dementia	29
Total		129		170

The harmonized Cognitively Normal (CN) category in the comparison with ADNI includes participants classified as Normal Controls (NC), Subjective Cognitive Decline (SCD), and Impaired Not SCD/MCI from the 1Florida ADRC cohort. The Impaired Not SCD/MCI category represents individuals with subtle cognitive complaints who did not satisfy formal criteria for mild cognitive impairment or dementia and therefore does not have a direct equivalent diagnostic category within ADNI. For cross-cohort harmonization, these participants were grouped with the cognitively unimpaired categories for more comparable diagnostic mapping between cohorts.

**FIGURE 8:**
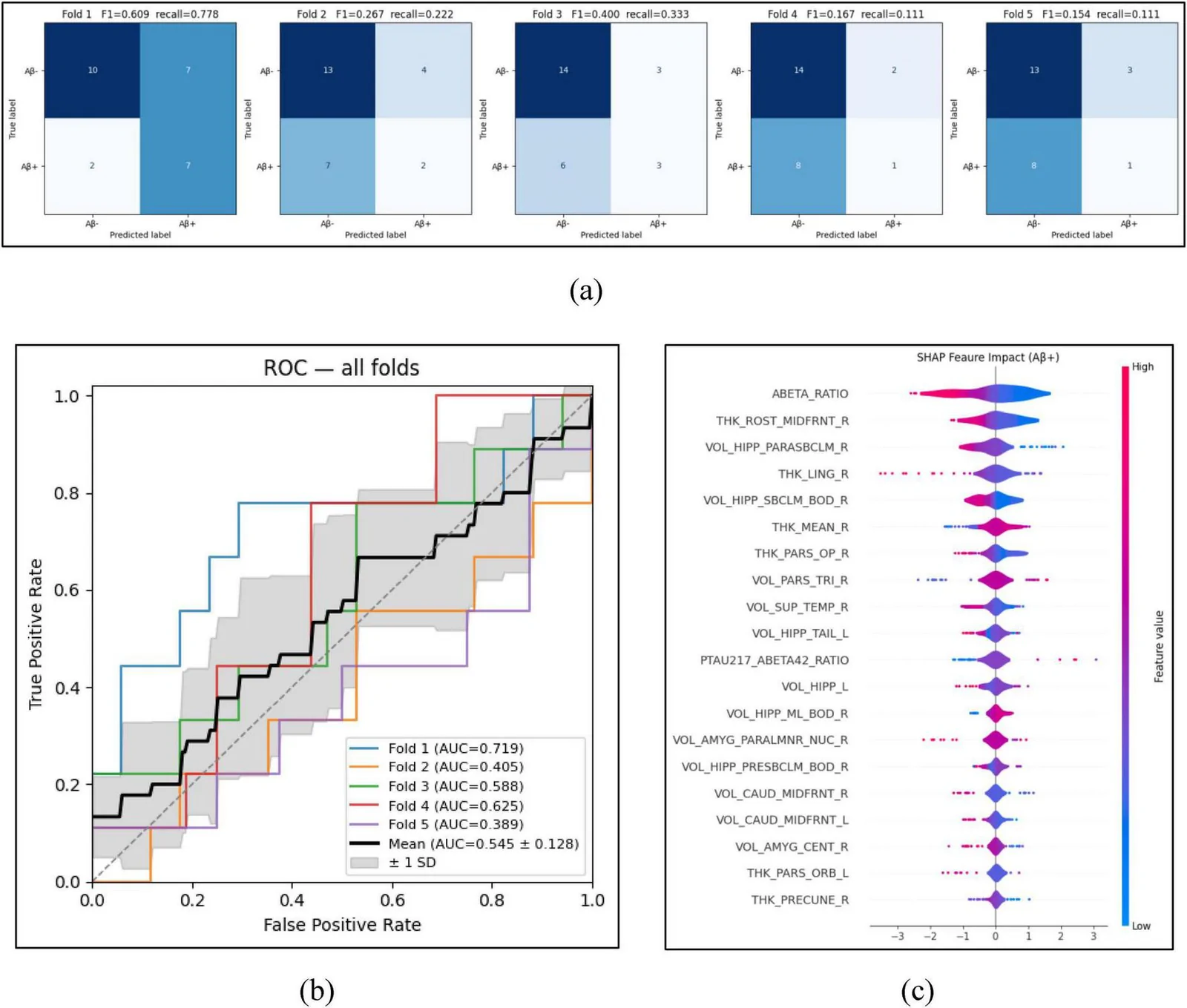
ML pipeline results within the ADNI4 dataset. **(a)** Confusion matrices per fold. **(b)** ROC curves per fold. **(c)** SHAP summary of feature impact on Aβ+ prediction.

The relatively high proportion of normal visual reads in ADNI4 also likely contributed to class imbalance, biasing predictions toward amyloid-negative cases. This interpretation is supported by the instability observed across folds seen in Figure 8a, indicating that the model was sensitive to how the ADNI4 data were partitioned even under stratified K-fold cross-validation. The mean AUC of 0.545 in Figure 8b suggests only slightly better-than-chance discrimination, which is consistent with limited separability between classes in this cohort. At the same time, the SHAP analysis in Figure 8c showed that the plasma biomarker ratio remained among the top predictive features, supporting the relevance of blood biomarkers for amyloid classification despite the weaker overall performance.

In retrospect, when trained and evaluated exclusively on ADNI4 using stratified cross-validation, the model’s performance was limited (mean AUC = 0.545), suggesting reduced class separability in this cohort. However, when the model was trained on the ADRC cohort and externally tested on ADNI4, discrimination improved to an AUC of 0.636, with an overall classification accuracy of 63%. Although performance remained lower than during internal ADRC validation, these results demonstrate that the framework captured biologically relevant signals that partially transferred between independent populations.

### ADNI4 external validation

**FIGURE 9:**
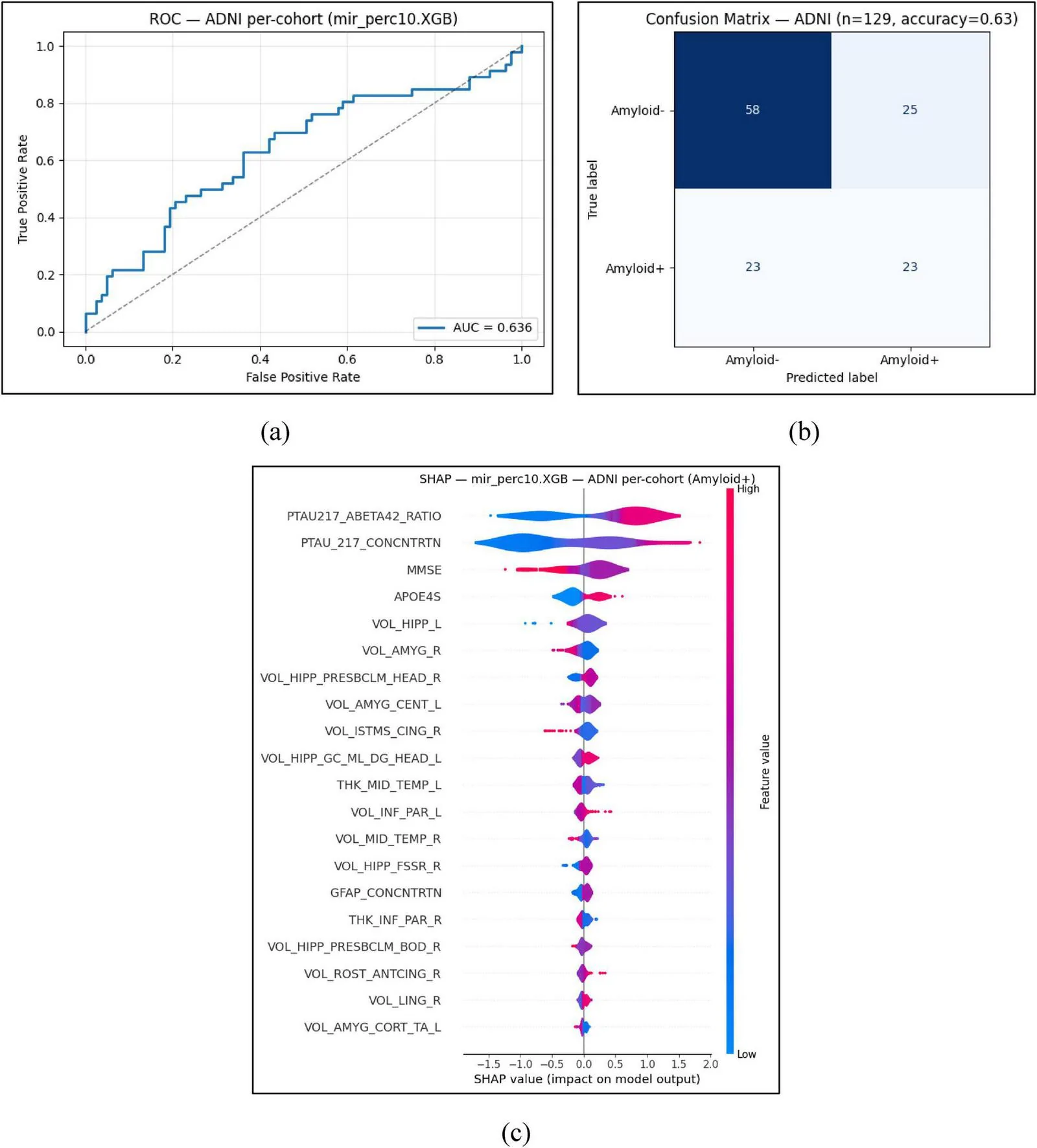
Cross-cohort evaluation with 1FL ADRC as the training set and ADNI4 as the test set. **(a)** ROC curve. **(b)** Confusion matrix. **(c)** SHAP summary for ADNI4, ordered by the 1FL ADRC ranking.

External validation was performed using data obtained from the Alzheimer’s Disease Neuroimaging Initiative 4 (ADNI4), a longitudinal multicenter study designed to develop clinical, imaging, genetic, and biomarker measures for Alzheimer’s disease research (Weiner et al., 2017). Because the ADNI4 cohort experiment did not yield adequate performance, we next trained the model on the 1FL ADRC cohort and tested it on ADNI4. Since the two cohorts differed substantially in their distributions, standardization was performed separately to prevent the 1FL ADRC from influencing the ADNI4 feature space. When this step was not applied, the model tended to classify nearly all participants as amyloid-negative, indicating a strong cohort shift between the two datasets. Using this cross-cohort approach, the model achieved an AUC of 0.636, as shown in Figure 9a, which was higher than the mean AUC obtained when the pipeline was trained and evaluated within ADNI4 alone. However, the performance remained modest, as seen in Figure 9b and Table 6, suggesting that the model demonstrated reduced generalizability.

**TABLE 6:** ADNI4 cohort classification report using 1FL ADRC as the training dataset.

Amyloid status and metrics	Precision	Recall	F1-score
Amyloid −	0.72	0.70	0.71
Amyloid +	0.48	0.50	0.49
Accuracy			0.63
Macro avg	0.60	0.60	0.60
Weighted avg	0.63	0.63	0.63

This limited transferability is likely due to the same structural differences discussed earlier, including differences in participant composition, cognitive stage distribution, and amyloid status balance between 1FL ADRC and ADNI4. In 1FL ADRC, the biomarker and diagnostic separation appear stronger, whereas ADNI4 contains a larger proportion of cognitively normal and amyloid-negative cases, which may shift the prediction distribution toward lower-risk outcomes. The SHAP analysis still showed that the p-tau217/Aβ42 ratio and p-tau217 concentration, Figure 9c, where the most influential features support the biological relevance of the blood markers, but not enough to fully overcome the cohort mismatch. Overall, these results suggest that the pipeline captured meaningful biomarker signals, but differences in cohort structure limited its portability.

## Discussion

This study developed and evaluated a multimodal machine-learning framework to predict amyloid-PET positivity from plasma biomarkers, cognition, demographic characteristics, APOE genotype, and MRI-derived structural features. Across all evaluated pipelines, nonlinear tree-based methods consistently outperformed linear and distance-based classifiers, indicating that amyloid prediction benefits from flexible modeling of complex interactions among biomarkers and imaging variables. The best-performing pipeline, based on XGBoost with mutual-information feature selection, demonstrated strong classification performance with high recall and robust cross-validation AUC values, supporting the feasibility of multimodal machine-learning approaches for identifying amyloid pathology.

One of the most important findings was the dominant contribution of plasma biomarkers to model performance. SHAP analyses consistently identified the p-tau217/Aβ42 ratio and plasma p-tau217 concentration as the strongest predictors of amyloid positivity. These findings agree with growing evidence suggesting that plasma p-tau217 is among the most accurate blood-based biomarkers for identifying Alzheimer’s disease pathology and may approach the diagnostic performance of more invasive or expensive modalities such as CSF analysis and amyloid PET imaging (Hansson et al., 2023; Karikari et al., 2020; Palmqvist et al., 2020). This is followed by p-tau181 and MMSE. These findings are highly consistent with emerging evidence demonstrating that plasma phosphorylated tau biomarkers strongly correlate with cerebral amyloid accumulation and Alzheimer’s disease pathology.

Another important finding for external validation was the reduction in predictive performance observed using the ADNI4 cohort. Such performance degradation is commonly observed when machine-learning models are transferred across independent populations and reflects the well-recognized challenge of dataset shift, whereby differences in demographics, disease prevalence, recruitment criteria, and biomarker distributions alter model behavior (Varoquaux and Cheplygina, 2022; Wen et al., 2020). The marked differences in diagnostic composition and amyloid prevalence between the ADRC and ADNI4 cohorts likely contributed to the observed decline in AUC.

These observations and the discordant cases from the error analysis reflect two distinct sources of heterogeneity that operate at different levels and call for different interpretations. Population-level heterogeneity operates between cohorts at the level of sampling. The ADRC and ADNI4 populations differed in cognitive-stage composition, with cognitively normal participants making up 32.4% of the development sample but 66.7% of the validation sample. They also differed in ancestry and ethnicity, with the ADRC cohort roughly 48% Hispanic White and 3% Black African American versus roughly 13% Hispanic White and 25% Black African American in ADNI4, and the biomarker features came from two different assay platforms, Simoa/ALZpath and Fujirebio Lumipulse. These findings illustrate population-level dataset shift, a property of who was sampled and how they were measured rather than of any individual participant. Individual-level neurobiological heterogeneity operates within a single cohort at the participant level.

As the Centiloid-anchored error analysis shows, two individuals with the same binary visual-read label can carry markedly different plasma and structural profiles, with several misclassified cases falling in the 10—-30 CL intermediate zone or disagreeing with the visual read despite clearly elevated burden (Asken et al., 2024; Guevara et al., 2026; Iaccarino et al., 2025). This heterogeneity is not reducible to sampling and would persist even in a perfectly representative cohort, because it arises from imposing a coarse binary label on a continuous biology. The two mechanisms are distinct but compounding, and they call for different remedies. Broader, more representative, assay-harmonized training data addresses the population-level shift. It does not address the individual-level heterogeneity, which instead argues for models that emit calibrated probabilistic or Centiloid-anchored outputs rather than a hard binary decision, so borderline cases are represented rather than forced into a class. Distinguishing the two clarifies both why the model degraded across cohorts and what a clinically deployable version would require.

Although plasma biomarkers carried the strongest predictive signal, additional modalities contributed complementary information, thereby refining classification performance. APOE ε4 status emerged as a leading contributor, consistent with its established role as a major genetic risk factor for amyloid accumulation and Alzheimer’s disease progression. Structural MRI variables contributed more modestly but still provided meaningful contextual information, suggesting that neurodegenerative patterns may help refine biomarker-driven predictions. Similarly, cognitive measures such as MMSE contributed to the model despite the dominant influence of biological biomarkers, indicating that clinical expression remains informative when integrated with molecular and imaging data.

The use of fold-wise feature selection and cross-validation was an important methodological strength of this study. Feature selection was performed independently within each training fold, preventing information leakage from the held-out test partitions and improving the validity of performance estimates. In addition, Bayesian hyperparameter optimization enabled efficient exploration of classifier-specific parameter spaces while remaining computationally feasible. Together, these design choices strengthened the robustness and reproducibility of the evaluation framework.

A notable aspect of this work was the use of SHAP explainability analyses to investigate both global and individual prediction behavior. Global SHAP rankings demonstrated that the same plasma biomarkers consistently dominated model predictions across folds, whereas local explanations revealed that prediction outcomes reflected integrated interactions among biomarkers, cognition, APOE status, and MRI measures. Importantly, the error analysis showed that false-positive and false-negative predictions often arose when opposing modality-specific signals competed within individual participants. These findings highlight the complexity of amyloid classification and emphasize the importance of explainable artificial intelligence approaches for understanding how multimodal information contributes to prediction decisions. Thus, the consistency of SHAP rankings across cohorts strengthens confidence that the model learned biologically meaningful relationships rather than cohort-specific artifacts (Varoquaux and Cheplygina, 2022). Explainability frameworks are increasingly recognized as essential for clinical deployment because they enable verification of model reasoning and facilitate clinician trust in AI-assisted decision support systems (Holzinger et al., 2019; Lundberg and Lee, 2017).

The findings also have potential clinical implications. Blood-based biomarkers are increasingly viewed as scalable alternatives to PET imaging for screening individuals at risk for Alzheimer’s disease pathology. The strong predictive performance observed in this study suggests that multimodal machine-learning systems integrating plasma biomarkers with accessible clinical information may help reduce reliance on expensive imaging procedures, particularly in large-scale screening settings or resource-limited environments. Furthermore, explainable models may improve clinician confidence and facilitate integration into precision medicine workflows by providing interpretable decision support rather than black-box classifications. These findings further support recommendations advocating for the integration of blood-based biomarkers into Alzheimer’s disease diagnostic workflows, perhaps as an initial screening strategy before seeking confirmation through PET imaging or cerebrospinal fluid testing (Barthélemy et al., 2024; Jack et al., 2024; Teunissen et al., 2022).

Several limitations should be acknowledged. The development cohort was modest in size, and external validation required KNN imputation for predominantly missing GFAP and NfL measurements. Although imputation improved external-validation performance compared with complete-case analysis, uncertainty remains. In addition, ADRC and ADNI4 used different immunoassay platforms (Simoa/ALZpath versus Fujirebio Lumipulse), producing systematic differences in biomarker scales that likely contributed to reduced cross-cohort performance independently of biological variability. Differences in ancestry, ethnicity, cognitive-stage composition, and assay platform represent distinct sources of variability that future multicenter studies should address through larger, more representative cohorts and assay harmonization. Longitudinal modeling approaches could likewise offer insight into temporal disease progression and conversion risk beyond cross-sectional amyloid classification.

## Conclusion

This study presents an explainable multimodal machine-learning framework for predicting amyloid PET positivity by integrating plasma biomarkers, cognitive assessments, APOE genotype, demographic information, and structural MRI features. Across all evaluated models, plasma biomarkers, particularly the p-tau217/Aβ42 ratio and plasma p-tau217, emerged as the dominant predictors of amyloid pathology, while MRI and cognitive measures provided complementary information that improved predictive performance and biological interpretability.

Beyond its predictive accuracy, this work demonstrates that explainable artificial intelligence can provide meaningful insight into the biological basis of model decisions. The incorporation of SHAP analyses together with Centiloid-informed error analysis showed that several apparent misclassifications occurred in participants occupying intermediate or biologically complex positions along the continuous amyloid spectrum rather than representing simple algorithmic failures. These findings emphasize the importance of distinguishing population-level heterogeneity, which influences model generalizability across cohorts, from individual-level neurobiological heterogeneity, which reflects the continuous and variable nature of Alzheimer’s disease pathology.

Although external validation revealed reduced performance due to differences in cohort composition, demographic diversity, biomarker acquisition, and assay platforms, the preservation of biologically meaningful feature importance across cohorts supports the robustness and interpretability of the proposed framework. Future studies involving larger, multi-center, and demographically diverse populations with harmonized biomarker measurements and quantitative amyloid metrics will be essential to further improve generalizability and clinical applicability.

Overall, our findings support explainable multimodal machine-learning frameworks as clinically interpretable decision-support tools that complement blood-based biomarkers for scalable Alzheimer’s disease screening. By integrating predictive performance with biological interpretability, this approach has the potential to facilitate earlier identification of individuals at risk for Alzheimer’s disease while providing greater confidence in AI-assisted decision-making.

## Data Availability

The data analyzed in this study is subject to the following licenses/restrictions: For ADNI dataset users must complete a quick online registration and approval process and must sign the ADNI Data Use Agreement (DUA). For the 1FL ADRC dataset, a request needs to be made to the executive committee 1FL ADRC. Requests to access these datasets should be directed to https://adni.loni.usc.edu/, https://1floridaadrc.org/.
